# Carbamylated erythropoietin attenuates cardiomyopathy via PI3K/Akt activation in rats with diabetic cardiomyopathy

**DOI:** 10.3892/etm.2013.1134

**Published:** 2013-05-31

**Authors:** HONGYING HE, XIAOYU QIAO, SUISHENG WU

**Affiliations:** 1 Department of Geriatrics, The First Bethune Hospital of Jilin University, Changchun, Jilin 130021;; 2 Edmond H Fischer Signal Transduction Laboratory, College of Life Sciences, Jilin University, Changchun, Jilin 130012, P.R. China

**Keywords:** carbamylated erythropoietin, diabetes, cardiomyopathy, cell signal transduction, phosphatidylinositol-3-kinase/Akt, apoptosis

## Abstract

The aim of the present study was to investigate the protective effect of carbamylated erythropoietin (CEPO) against cardiomyopathy in high-fat, high-carbohydrate diet-fed rats with streptozotocin (STZ)-induced diabetic cardiomyopathy (DCM). Healthy male Wistar rats were fed a high-fat, high-carbohydrate diet for four weeks, and then were injected with STZ twice (50 mg/kg, intraperitoneally). Once DCM was confirmed, the rats were divided randomly into the following groups: DCM without treatment, CEPO treatment at different dosages (500, 1,000 or 2,000 IU/kg) or recombinant human erythropoietin (rhEPO) treatment (1,000 IU/kg), for a four-week short intervention or an eight-week long intervention protocol. Healthy rats were used as normal controls. Venous blood samples were drawn for routine hematological examinations, and heart tissues were collected for histological analysis, as well as the determination of myocardial apoptosis and phosphatidylinositol-3-kinase (PI3K)/Akt signaling. CEPO treatment had no significant effect on the erythrocyte or hemoglobin levels in the rats with DCM; however, it reduced myocardial cell apoptosis in the rats and protected the cellular ultrastructure. In addition, CEPO treatment inhibited caspase-3 and increased Bcl-xl protein expression (P<0.05). It also increased PI3K (p85) and Akt1 expression at the mRNA and protein levels in the hearts of the rats with DCM, with a dose-response relationship. An eight-week treatment using CEPO, in comparison with a four-week protocol, marginally increased PI3K (p85) and Akt1 expression, and did not demonstrate significant benefit. The study indicated that CEPO protects against DCM, without markedly affecting erythropoiesis, and that the activation of PI3K/Akt may be a key mechanism in the protection conferred by CEPO.

## Introduction

Diabetic cardiomyopathy (DCM) is a unique cardiovascular disease, which is characterized by myocardial relaxation and contractional dysfunction, as a result of oxidative stress, inflammation, cardiac fibrosis and myocardial apoptosis (1). During previous decades, there has been a particular focus on the role of apoptosis, i.e., the programmed cell death that is controlled by intrinsic genetic mechanisms in certain physiological or pathological conditions, in the pathogenesis of DCM.

Erythropoietin (EPO) is a glycoprotein hormone secreted by the kidney in the adult, and by the liver in the fetus, that stimulates the production of red blood cells by stem cells in the bone marrow (erythropoiesis). Synthetic products of EPO, such as recombinant human EPO (rhEPO), have been successfully applied in clinical practice to treat anemia induced by diabetic nephropathy (2). In addition, rhEPO has a variety of cellular protective effects, predominantly due to its binding to a heterodimer formed by the subunits of the EPO receptor (EPOR) and the β common receptor (βcR, also known as CD131). CD131 expression has been exhibited in the brain, heart, liver and kidney (3). When EPO binds to the EPOR-CD131 heterodimer, it activates various signaling pathways involved in cell survival, metabolism and apoptosis (3,4). However, the long-term, high-dose application of rhEPO is likely to cause a variety of adverse effects, including hypertension and thrombogenesis, due to its effect on the blood hematocrit (5).

Carbamylated EPO (CEPO), a carbamoyl derivative of EPO, has similar pharmacokinetic characteristics to EPO (6,7). Moreover, it has been demonstrated that whilst CEPO does not stimulate the production of red blood cells, it does bind to the EPOR-CD131 heterodimer to exert cellular protective effects similar to those of EPO (8–11). CEPO may limit mitochondrial permeability transition pore opening and prevent the release of mitochondrial contents, such as reactive oxygen species and cytochrome *c*, thereby reducing apoptosis (5).

In addition, CEPO affects cellular signal transduction, via the extracellular signal-regulated kinase (ERK)-1/2 (12) and Akt (13) pathways. However, to date, it has been unclear whether CEPO has a protective effect against myocardial apoptosis in rats with DCM. In this study, we investigated the effects of CEPO treatment on the myocardial apoptosis and phosphatidylinositol-3-kinase (PI3K)/Akt gene expression in the hearts of high-fat, high-carbohydrate diet-fed rats with streptozotocin (STZ)-induced DCM.

## Materials and methods

### 

#### Reagents

The rhEPO (cat. no. E10–053) was obtained from Kai Mao Biomedical Co., Ltd. (Shanghai, China), and STZ (cat. no. S0130), diethyl pyrocarbonate (DEPC), sodium borate and potassium cyanate were purchased from Sigma-Aldrich (St. Louis, MO, USA). The terminal deoxynucleotidyl transferase-mediated dUTP nick end labeling (TUNEL) kit (*In Situ* Cell Death Detection kit, POD) was obtained from Roche Diagnostics, Basel, Switzerland. The caspase-3 (cat. no. 9662), Bcl-xL (54H6; cat. no. 2764), p-PI3K (p85; cat. no. 4292) and p-Akt [serine/threonine (Ser/Thr); cat. no. 9611s] antibodies were purchased from Cell Signaling Technology, Inc. (Danvers, MA, USA). The PI3K (p85α) and Akt1 mRNA *in situ* hybridization kits were obtained from Tianjin Haoyang Biological Manufacture Co., Ltd. (Tianjin, China). All the reagents were of analytical pure grade.

#### CEPO synthesis

CEPO was synthesized as previously described by Leist *et al* (7). In brief, potassium cyanate was added to a mixture of 500 *μ*l rhEPO (1 mg/ml) and 500 *μ*l 1M sodium borate, to produce a solution with a final concentration of 1 mol/l. This was mixed and incubated at 37°C for 24 h to form the reaction solution. Excess potassium cyanate was removed from the reaction solution by dialysis, and the solution was then further concentrated by ultrafiltration (membrane cut-off, 10 kDa). The protein concentration was measured by Coomassie Brilliant Blue staining, and the absorbance was read at 335 nm.

### Animals

#### Establishment of the DCM rat model

Healthy male Wistar rats (n=120, including 20 as controls and 100 for the DCM model; weight, 220±20 g), and the high-fat, high-carbohydrate diet (comprising 66.6% basic rat chow, 20% sucrose, 10% lard, 3% egg yolk and 0.4% cholesterol) were obtained from the Experimental Animal Center of Jilin University (Changchun, China). The study was approved by the ethics committee of The First Bethune Hospital of Jilin University. In order to establish the diabetic model, 100 rats were fed a high-fat, high-carbohydrate diet for four weeks, and were then injected with STZ (50 mg/kg, intraperitoneally). A second injection of the same dose of STZ was administered a week later. Seventy-five rats were identified to have diabetes mellitus, according to the criterion of a fasting blood glucose concentration >18 mmol/l, and were used in the following experiments. The control group was fed with a normal diet.

#### Animal grouping

The experiment was divided into two studies, in order to evaluate the dose- and time-dependent responses to CEPO administration. In study one, which analyzed the dose-response relationship, the rats were assigned to the following groups: Control (group A, healthy rats, n=10); DCM (group B, n=9); CEPO (500 IU/kg; group C, n=9); CEPO (1,000 IU/kg; group D, n=9), CEPO (2,000 IU/kg; group E, n=9) and rhEPO (1,000 IU/kg; group F, n=10), where groups B-F comprised rats with DCM. The CEPO or rhEPO was dissolved in 0.3 ml physiological saline, and then subcutaneously injected twice a week for four weeks. The final numbers of surviving rats in each group were 10, 6, 7, 7, 8 and 7, respectively. Study two, a time-response relationship analysis, included a short-term (four-week) intervention panel, which comprised groups A, B, D and F, as above, and a long-term (eight-week) intervention panel, which comprised the following groups: Control (group A′, healthy rats, n=10); DCM (group B′, n=9); CEPO (1,000 IU/kg; group D′, n=9) and rhEPO (1,000 IU/kg; group F′, n=10). The final numbers of surviving rats in each group of the long-term intervention were 10, 6, 8 and 7, respectively.

### Assays for blood samples or heart tissues

#### Routine blood examination

Rats from each group were fasted for 8 h and anesthetized with 10% chloral hydrate (0.30 g/kg), following the intervention protocol. Blood samples were collected with red-top normal serum and purple EDTA anticoagulation vacutainer tubes from the right ventricles of six randomly selected rats from each group, and were then sent for routine hematological examination at the clinical laboratory of The First Bethune Hospital of Jilin University (Changchun, China), using a leukocyte five-part differential hematology analyzer.

#### Myocardial cell transmission electron microscopy (TEM)

Eight weeks following the commencement of the experiment, the rat hearts from each group were perfused with 37°C 0.9% NaCl, along with a 4°C paraformaldehyde and 4% glutaraldehyde mixture. The heart apices (∼2 mm) were processed successively with 2.5% glutaraldehyde and 1% osmic acid fixations, ethanol series dehydration and Epon 812 epoxy resin embedding, and were then sectioned with an LKB 8800 Ultratome III (Bromma, Sweden). Uranyl acetate and lead citrate double staining, and a JEM-1200EX transmission electron microscope were used to observe the sections, and photographic images were captured.

#### TUNEL investigation of myocardial cell apoptosis in the rats

Eight weeks following the commencement of the experiment, the rat hearts from each group were perfused and fixed with 10% neutral buffered formalin, and then paraffin-embedded. Sections measuring 4 *μ*m were used for the detection of myocardial cell apoptosis with the TUNEL kit (Roche Diagnostics), in accordance with the manufacturer’s instructions. Apoptosis was quantified using Image-Pro Plus 6.0 image analysis software (Media Cybernetics, Rockville, MD, USA), in order to facilitate quantitative analysis.

#### Immunohistochemistry

The paraffin-embedded heart sections were examined for caspase-3 (1:200) and Bcl-xl (1:300) protein expression, using a streptavidin-peroxidase (SP) assay. Cells were defined as positive if the cytoplasm was stained brown. The integrated optical density (IOD) of the positively-stained tissue was calculated using Image-Pro Plus 6.0 software (Media Cybernetics).

#### In situ hybridization for determination of PI3K/Akt mRNA expression

The PI3K and Akt mRNA expression was determined by *in situ* hybridization assay, in accordance with the manufacturer’s instructions. The probe sequences used for the PI3K (p85α) mRNA were as follows: i) 5′-GTCTC CCCTC TCCCC AGTAG TTTCA TTG; ii) 5′-ATAAG GAGAG GCGGG GCAAC ATCAG GAG and iii) 5′-GTAAG TCGGC GAGAT AGCGT TTGAA AGC. The probe sequences used for the Akt1 mRNA were as follows: i) 5′-CCCTC CTTCA CAATG GCTAC GTCGT TCA; ii) 5′-GCTTC AGGTA CTCAA ACTCG TTCAT GGT and iii) 5′-TCTCA GTAAG CGTGT GGGCA ACCTC ATC. Cells were defined as positive if the cytoplasm was stained brown. The positive staining was quantified using the IOD, which was calculated by Image-Pro Plus 6.0 software (Media Cybernetics).

#### Western blot analysis of PI3K/Akt phosphorylated protein expression

A small quantity of myocardial tissue was cut into fragments, and then homogenized with radioimmunoprecipitation assay (RIPA) buffer (1mM phenylmethanesulfonyl fluoride) for protein extraction. Following the centrifugation of the lysates (13,750 × g for 15 min at 4°C), the supernatants were quantified using a bicinchoninic acid protein assay. The protein concentration in the loading samples was adjusted to 80 *μ*g/20 *μ*l, and then the protein samples were separated by 10% sodium dodecyl sulfate-polyacrylamide gel electrophoresis (SDS-PAGE) gel, and transferred to a polyvinylidene fluoride (PVDF) solid-phase membrane. The p-PI3K (p85) (1:1,000), p-Akt (Ser/Thr) (1:1,000) and β-actin (1:1,000) antibodies were incubated with the PVDF membranes for 90 min, and then the membranes were conjugated with a second anti-immunoglobulin (Ig)-G antibody (1:2,000) for 90 min. The immunoblotted proteins were subsequently detected using enhanced chemiluminescence.

#### Statistical analysis

All data are expressed as the mean ± standard deviation (SD). Statistical analysis was performed using SPSS software, version 13.0 (SPSS, Inc., Chicago, IL, USA). The difference between the means of two groups was assessed using the Student’s t-test, while multiple means were compared using one-way analysis of variance. P<0.05 was considered to indicate a statistically significant difference.

## Results

### 

#### Routine hematological examination

Compared with the control and DCM groups, CEPO had no effect on the red blood cell count, hematocrit or hemoglobin levels (Table I). However, following four weeks of treatment, rhEPO significantly increased the number of red blood cells and the hemoglobin level in the rats with DCM (P<0.05 for each), compared with their values in the control group, in addition to inducing a significantly higher hematocrit than that induced by CEPO (P<0.05). Eight weeks of treatment with rhEPO resulted in a higher hematocrit than four weeks of treatment (P<0.05).

#### TEM of heart tissues

**Figure 1.**
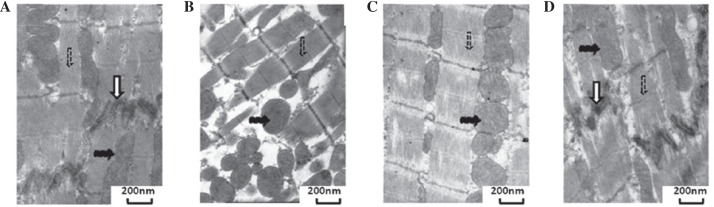
Transmission electron microscope images show that carbamylated erythropoietin (CEPO) protects myocardial cell ultrastructure in rats with diabetic cardiomyopathy (DCM). (A) Control, (B) DCM, (C) CEPO (1,000 IU/kg) and (D) recombinant human EPO (1,000 IU/kg) groups. The control group comprised healthy rats, whereas the rats of the other groups had DCM. Muscle fibers (dashed arrow), mitochondria (black arrow) and intercalated discs (white arrow) are shown. Uranyl acetate and lead citrate double-staining. Stained with uranyl acetate and lead citrate. Magnification: (A) ×20K; (B) ×12K; (C) ×20K; (D) ×20K.

TEM analysis of the myocardial cells demonstrated that in the control group, the I and A bands were clear, with visible transverse tubules on the level of the Z line. The mitochondria were oval in shape, and the ridge was closely spaced with the intact membrane. The gap junctions were visible and dense. In the DCM group, there was a reduction in the fibrin bundles in the cytosol, and a number of bundles had dissolved in the sarcomere, leading to matrix cavitation. The intercalated discs were moderately separated, and the gap junctions were reduced. There was an increase in the number of mitochondria, and several mitochondria were small, circular and pyknotic in appearance. In the CEPO group, the level of fibrin was reduced, and dissolution had occurred in small sections of the sarcomeres, although in general the I and A bands were clear. There was an increase in the number of mitochondria, a small number of which were small and circular in shape. In certain instances, the individual M lines and H bands were not clear, due to a mild swelling. In the rhEPO group, there was limited rupturing or dissolving of the fibrin in the sarcomere, the intercalated disc cross connections were clear, and there was only a lack of clarity in certain local structures of individual connections (Fig. 1).

#### Detection of myocardial cell apoptosis in rats with DCM

**Figure 2.**
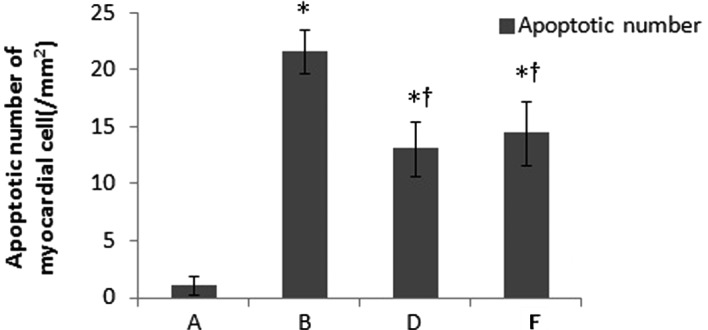
Number of apoptotic myocardial cells in different experimental groups. Group A, control; B, diabetic cardiomyopathy (DCM); D, carbamylated erythropoietin (CEPO; 1,000 IU/kg); and F, recombinant human EPO (1,000 IU/kg). ^*^P<0.05 vs. control and ^†^P<0.05 vs. the DCM group.

The results of the TUNEL assay demonstrated that the nuclei of the apoptotic cells were brown, whereas those of the normal cells were blue. There were a few, scattered apoptotic nuclei in the control group, whereas numerous apoptotic cells were observed in the DCM group. The number of apoptotic myocardial cells in the DCM group was identified to be 21.557±1.915/mm^2^, which was significantly higher than the number in the control group (P<0.05). In the CEPO group, the number of apoptotic myocardial cells was 13.083±2.371/mm^2^, which indicated a smaller increase than that in the DCM group (P<0.05), although the result remained higher than that in the control group (P<0.05). The rhEPO group demonstrated a similar result to that of the CEPO group, with the number of apoptotic myocardial cells observed to be 14.476±2.804/mm^2^ (Fig. 2).

#### Immunohistochemistry for caspase-3 and Bcl-xl

**Figure 3.**
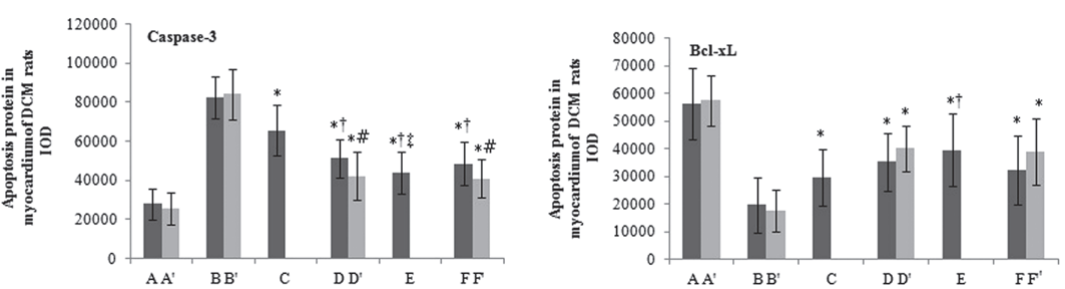
Effects of carbamylated erythropoietin (CEPO) on caspase-3 and Bcl-xl protein expression in the myocardial immunohistochemical examination of rats with diabetic cardiomyopathy (DCM). Rats were assigned to the following groups for a four-week treatment intervention (black bars): A, control; B, DCM; C, CEPO (500 IU/kg); D CEPO (1,000 IU/kg); E, CEPO (2,000 IU/kg); and F, recombinant human (rh)-EPO (1,000 IU/kg). For the eight-week treatment intervention (grey bars), the groups were as follows: A′, control; B′, DCM; D′, CEPO (1,000 IU/kg); and F′, rhEPO (1,000 IU/kg). Groups A and A′ comprised healthy rats, whereas groups B-F and B′-F′ comprised rats with DCM. ^*^P<0.05 vs. DCM, ^†^P<0.05 vs. CEPO (500 IU/kg), ^‡^P<0.05 vs. CEPO (1,000 IU/kg), ^§^P<0.05 vs. CEPO (2,000 IU/kg) groups and ^#^P<0.05 vs. the four-week treatment course group. IOD, integrated optical density.

The caspase-3 protein expression level in the rats with DCM was significantly increased to ∼4-fold that of the control group, whereas the Bcl-xl protein expression was significantly reduced in the four- and eight-week treatment groups. In comparison with the DCM group, the CEPO and rhEPO treatments significantly ameliorated the increase in caspase-3 and the reduction in Bcl-xl protein levels, exhibiting a dose-dependent relationship (P<0.05). Treatment with 1,000 IU/kg CEPO demonstrated a similar effect to the same dose of rhEPO on the amelioration of the caspase-3 and Bcl-xl protein expression in the diabetic hearts (P>0.05). Of note, the eight-week CEPO and rhEPO treatments demonstrated an enhanced effect on the suppression of caspase-3 protein expression, compared with the four-week treatments; however, no difference was observed in Bcl-xl protein expression between the four- and eight-week treatments (Fig. 3).

#### In situ hybridization for determination of PI3K/Akt mRNA expression

**Figure 4.**
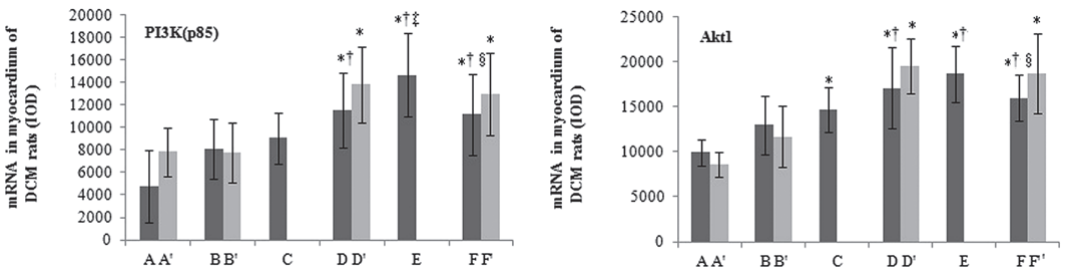
Effects of carbamylated erythropoietin (CEPO) on phosphatidylinositol-3-kinase (PI3K)/Akt mRNA expression (examined by *in situ* hybridization) in the myocardial tissues of rats with diabetic cardiomyopathy (DCM). Rats were assigned to the following groups for a four-week treatment intervention (black bars): A, control; B, DCM; C, CEPO (500 IU/kg); D CEPO (1,000 IU/kg); E, CEPO (2,000 IU/kg); and F, recombinant human (rh)-EPO (1,000 IU/kg). For the eight-week treatment intervention (grey bars), the groups were as follows: A′, control; B′, DCM; D′, CEPO (1,000 IU/kg); and F′, rhEPO (1,000 IU/kg). ^*^P<0.05 vs. DCM, ^†^P<0.05 vs. CEPO (500 IU/kg), ^‡^P<0.05 vs. CEPO (1,000 IU/kg) and ^§^P<0.05 vs. CEPO (2,000 IU/kg) groups. IOD, integrated optical density.

Compared with the control group, the levels of myocardial cell PI3K (p85α) and Akt1 mRNA expression were increased in the DCM group (P<0.05). CEPO treatment further increased the levels of PI3K (p85α) and Akt1 mRNA expression in the heart tissues of the diabetic rats (P<0.05 for each), and the increase was dose-dependent (P<0.05). No significant differences were observed in the levels of PI3K (p85α) and Akt1 mRNA expression between the CEPO and rhEPO groups that received the same dosage (P>0.05). There was a higher elevation in the levels of PI3K (p85α) and Akt1 mRNA expression in the hearts of the rats with DCM following eight weeks of treatment, as opposed to four-weeks, but the difference was not significant (Fig. 4).

#### Western blot analysis of PI3K/Akt phosphorylated protein expression

**Figure 5.**
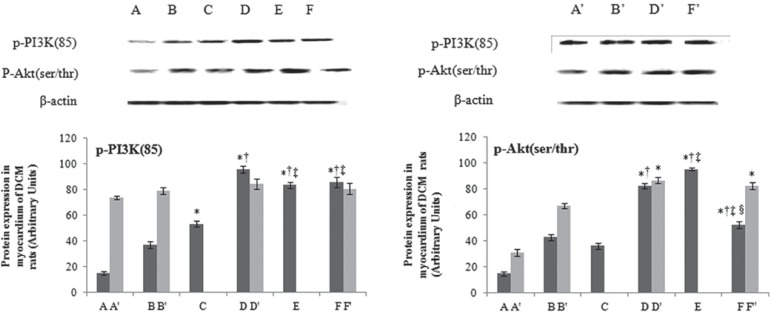
Effects of carbamylated erythropoietin (CEPO) on phosphatidylinositol-3-kinase (PI3K)/Akt protein expression (examined by western blot analysis) in the myocardial tissues of rats with diabetic cardiomyopathy (DCM). Rats were assigned to the following groups for a four-week treatment intervention (black bars): A, control; B, DCM; C, CEPO (500 IU/kg); D CEPO (1,000 IU/kg); E, CEPO (2,000 IU/kg); and F, recombinant human (rh)-EPO (1,000 IU/kg). For the eight-week treatment intervention (grey bars), the groups were as follows: A′, control; B′, DCM; D′, CEPO (1,000 IU/kg); and F′, rhEPO (1,000 IU/kg). ^*^P<0.05 vs. DCM, ^†^P<0.05 vs. CEPO (500 IU/kg), ^‡^P<0.05 vs. CEPO (1,000 IU/kg) and ^§^P<0.05 vs. CEPO (2,000 IU/kg) groups.

Similar to the mRNA expression, the p-PI3K (85) and p-Akt (Ser/Thr) protein expression levels in the myocardial cells of the rats with DCM were significantly increased, compared with those of the normal control rats (P<0.05). In comparison with the rats with DCM, CEPO treatment further increased the p-PI3K (p85) and p-Akt (Ser/Thr) protein expression levels in the myocardial cells, in a dose-dependent manner (P<0.05). There were no significant differences in the PI3K (p85) and p-Akt (Ser/Thr) protein expression levels between the same-dose CEPO and rhEPO groups (P>0.05). No significant difference was observed between the four- and eight-week treatment regimens (Fig. 5).

## Discussion

In this study we revealed that CEPO, a carbamoyl derivative of EPO that does not affect erythropoiesis, attenuated myocardial pathological damage, at least in part through the activation of the PI3K/Akt signaling pathway, which regulates myocardial cell apoptosis in diabetes mellitus.

It has previously been demonstrated that CEPO exerts a protective effect against apoptosis in central and peripheral nervous system (8,14,15), cardiovascular (16–18) and urinary system diseases (11,19,20), as well as in diabetic peripheral neuropathy-induced peripheral nerve muscular atrophy (21). However, whether CEPO is able to confer protection against DCM remains unknown.

CEPO and rhEPO exert their anti-apoptotic effects through their capacity to bind to EPOR-CD131, as EPO does (3,4). However, they differ in their effects on erythropoiesis. rhEPO binds to its classic receptor, (EPOR)_2_, and stimulates red blood cell proliferation and differentiation, under the regulation of hypoxia and vasoconstriction (14), which in turn is apt to result in an excessive hematocrit and thrombogenesis. CEPO does not bind to (EPOR)_2_, and therefore it does not increase the the risk of blood clots by causing the excessive production of red blood cells, even when it is used for long-term high-dose treatment, or in the case of severe hypoxia (7,12). In the present study, we revealed that the long-term use of CEPO did not cause an elevation in the number of red blood cells in rats with DCM. These results suggested that the application of CEPO may reduce the risk of adverse effects, including thrombosis and high blood pressure.

In the current study we used healthy male Wistar rats, fed with a high-fat, high-carbohydrate diet, and treated with STZ injections, to successfully generate rats with diabetes mellitus. We used a TUNEL assay and TEM to observe myocardial cell apoptosis, and demonstrated that CEPO and rhEPO protected myocardial cells from apoptosis by reducing apoptotic rates and by improving cell ultrastructure. These results were consistent with the findings of previous studies (4,5).

Cell apoptosis is regulated through the balance between pro- and anti-apoptotic proteins. Among those, caspase-3, which belongs to the cysteinyl aspartate-specific protease (caspase) family, is vital in initiating the process of apoptosis. In the diabetic condition, high blood glucose-induced oxidative stress activates the mitochondrial cytochrome *c*-mediated caspase-3 pathway, resulting in myocardial cell apoptosis (22). Elevated caspase-3 expression has been observed in the myocardial tissues of a number of pathological conditions, including diabetes (23,24), diabetic myocardial ischemia (25), myocardial infarction (26) and ischemia/reperfusion injury (27), leading to increased apoptosis. The inhibition of caspase-3 expression may confer protection against diabetes-induced myocardial apoptosis (28). By contrast, Bcl-xl, which belongs to the B cell lymphoma/leukemia-2 (Bcl-2) family, is considered to be one of the major anti-apoptotic proteins. Bcl-xl is a dominant subtype of the Bcl-x (Bcl-211) gene-encoding products (29), and its structure is similar to that of Bcl-2. Bcl-xl has been demonstrated to be important in protecting against the apoptosis of cardiomyocytes (30). In the current study, the expression level of caspase-3 protein was increased, and that of Bcl-xl protein was decreased in rats with DCM, compared with the levels in the control group. CEPO treatment significantly downregulated caspase-3 and upregulated Bcl-xl protein expression, suggesting that CEPO has protective effects against apoptosis in DCM myocardial cells. The binding of EPO to EPOR-CD131 affects multiple signal transduction pathways, including the PI3K/Akt pathway, which is crucial for insulin signal transduction, the cell cycle, and cell growth and survival (31). PI3K is an enzyme complex consisting of a regulatory subunit, p85, and a catalytic subunit, p110. There are five subtypes of the regulatory subunit: p85α and -β, p55γ and -α, and p50α. Among these, p85α is the sole subtype to be correlated with the glucose transporter 4 (31,32). The activation of PI3K subsequently activates its downstream Ser/Thr protein kinase, Akt, also known as protein kinase B. When Ser 308 and Thr 473 of Akt are phosphorylated, Akt is activated (33). The phosphorylation of Akt (p-Akt) further leads to the phosphorylation of Ser 196 of caspase-9, and Ser 136 of Bcl-2-associated death promoter protein, which deprives them of pro-apoptotic effects (34). High blood glucose, which leads to myocardial oxidative stress, activates numerous inflammatory cytokines, and induces myocardial apoptosis through the Akt pathway (35). Myocardial atrophy in rats with DCM is correlated with impaired Akt phosphorylation (36). In the current study, CEPO increased PI3K (p85α) and Akt1 mRNA expression levels, and also enhanced p-PI3K (p85) and p-Akt (Ser/Thr) protein expression levels in the hearts of rats with DCM, in a dose-dependent manner. This suggested that CEPO may exert anti-apoptotic effects in DCM through the activation of the PI3K/Akt pathway, as rhEPO does. Notably, although CEPO demonstrated a dose-dependent effect on DCM in this study, the long-term (8-week) treatment of CEPO failed to demonstrate a significant increase in PI3K (p85) and Akt1 mRNA expression, and did not demonstrate a significant benefit, when compared with the short-term (four-week) treatment. Therefore, a time-dependent response of DCM to CEPO was not established.

In conclusion, CEPO exhibited myocardial protection, without adverse effects on erythropoiesis, in rats with DCM, at least in part through the activation of the PI3K/Akt pathway. However, this study was based on animal models *in vivo*, and detailed molecular mechanisms should be further investigated using *in vitro* studies. Clinical trials may also be considered to validate the results in humans, following further pre-clinical studies.

## Figures and Tables

**Table I.** Effects of CEPO on the erythrocyte and hemoglobin levels in rats with DCM.

	Treatment duration (weeks)	No.	Group			
			A	B	D	F
RBC (10^12^/l)	4	6	6.97±0.66	8.19±0.59	8.77±0.37	9.96±0.28^a^
	8	6	7.05±0.64	8.30±0.13	8.02±0.35	10.44±0.30^a–c^
HGB (g/l)	4	6	142.5±26.60	150.5±12.02	153.0±12.73	187.5±4.95^a^
	8	6	144.5±14.85	154.0±1.41	149.5±10.61	195.5±7.78^a–c^
HCT (fl)	4	6	0.51±0.03	0.46±0.04	0.44±0.03	0.55±0.01^c^
	8	6	0.51±0.07	0.49±0.02	0.46±0.04	0.63±0.01^b–d^

CEPO, carbamylated erythropoietin; DCM, diabetic cardiomyopathy; no., number of rats; RBC, red blood cell; HGB, hemoglobin; HCT, hematocrit; group A, control; group B, DCM; group D, CEPO (1,000 IU/kg); group F, recombinant human EPO (1,000 IU/kg). Group A comprised healthy rats, whereas groups B,D and F comprised rats with DCM. Data are expressed as the mean ± standard deviation.

a P<0.05 vs. control;

b P<0.05 vs. DCM;

c P<0.05 vs. CEPO (1,000 IU/kg) and

d P<0.05 vs. the corresponding four-week intervention group.
